# Effect of a Fiber Raw Material Structure on the Properties of Cellulose/Polyacrylamide Double-Network Antifreezing Conductive Hydrogels Prepared in an AlCl_3_/ZnCl_2_ Aqueous System

**DOI:** 10.3390/gels12100865

**Published:** 2026-09-24

**Authors:** Xiaowei Zhuang, Shuqi Guo, Yue Liu, Zhijun Wu, Yongshun Feng, Hui Qiao, Guoqiang Zhu, Xin Pan

**Affiliations:** 1 Zhejiang Academy of Forestry, Liuhe Road 399, Hangzhou 310023, China; zhuangxiaowei@zjforestry.ac.cn (X.Z.);; 2 College of Environmental Science and Engineering, Liaoning Technical University, Zhonghua Road 47, Fuxin 123000, China

**Keywords:** cellulose, antifreezing, conductive hydrogel, ionic conductivity

## Abstract

The trade-off between mechanical properties and ionic conductivity, together with low-temperature failure, restricts the practical application of ionically conductive hydrogels. In this work, four bleached pulp boards (eucalyptus, pine, bagasse and cotton) were dissolved in an AlCl_3_/ZnCl_2_ aqueous solution, followed by in situ polymerization of acrylamide to fabricate cellulose/polyacrylamide double-network conductive hydrogels. The cotton-based gel exhibited the best pristine tensile performance (105 kPa, 1566%) and the highest room temperature ionic conductivity (50.5 mS/cm). At −30 °C, except for pure PAM (which froze beyond the load cell limit), the other five gels remained soft (stress at 70% strain only 1.13–1.35 times that at room temperature). After freeze–thaw cycles, the tensile strengths of the bagasse and pine gels increased by 52% and 46%, respectively, whereas that of the cotton-based gel decreased by 37%. At −40 °C, the pine-based gel retained the best conductivity (0.83 mS/cm, 5.1% retention). In addition, the gels exhibited strain-dependent electrical resistance responses at the material level. The structure–property correlations indicate that the influence of the fiber source does not depend solely on the degree of polymerization, but arises from the combined effects of the aggregation state of regenerated cellulose, pore wall architecture, dynamic hydrogen bonding and water-binding capability on ion channel continuity, low-temperature ion transport and freeze–thaw-induced network reorganization. This study provides a material-level experimental basis for selecting fiber raw materials for antifreezing conductive hydrogels. Device-level performance remains to be validated.

## 1. Introduction

Ionically conductive hydrogels have attracted considerable attention in the field of flexible ionotronics because of their tissue-like softness and tunable ion transport capability [1]. Unlike conventional conductors that rely on electron migration (e.g., carbon-based and metallic materials), ionically conductive hydrogels transmit signals through the directional migration of free ions [2,3]. This ion-based (rather than electron-based) conduction mechanism enables hydrogels to maintain stable signal transmission under large deformation, and the electrochemical effects accompanying ionic conduction provide ample room for developing novel ionotronic devices [4]. In recent years, conductive hydrogels have shown broad application prospects in wearable sensors, human–machine interaction, and soft robotics [5]. In energy storage fields such as aqueous zinc-ion batteries, gel electrolytes combining high mechanical strength with low-temperature adaptability are likewise regarded as an important direction for replacing liquid electrolytes [6].

However, the practical application of such materials has long been constrained by two major bottlenecks. The first is the inherent trade-off between mechanical properties and ionic conductivity [7,8,9]. The mechanical strength of a hydrogel generally relies on the crosslinking density and interchain interactions of the polymer network, whereas ion transport efficiency depends heavily on network porosity and segmental mobility. Increasing crosslinking points or introducing rigid components can enhance mechanical strength, but often leads to network contraction and reduced porosity, increasing the migration resistance of charge carriers and thereby decreasing conductivity [7,8]. This seesaw relationship between strength and conductivity has long been a central challenge in the design of high-performance conductive hydrogels [9,10]. The second bottleneck is performance failure at subzero temperatures [11,12]. Conventional hydrogels contain large amounts of free water; at subzero temperatures, ice-crystal nucleation and growth block ion transport pathways with solid ice, causing a sharp drop in conductivity, while the volumetric expansion of ice-crystals exerts local stress on the polymer network, leading to pore wall rupture and network collapse [13]. Such structural damage is often irreversible during freeze–thaw cycles and ultimately results in functional failure of the material [10,12]. The simultaneous resolution of these two bottlenecks is a key prerequisite for the practical application of ionically conductive hydrogels.

In recent years, researchers have attempted to break through the above bottlenecks from multiple directions. For example, constructing soft–hard microphase-separated structures can partially alleviate the strength–conductivity trade-off [7]; Hofmeister-effect-driven network reconstruction improves the ion transport and mechanical behavior of cellulose-based gels [8]; synergistic ionic and hydrogen bond locking strategies enhance the wide-temperature adaptability of gel electrolytes [14]; low-tortuosity ion channel designs accelerate ion migration [15,16]; metal-coordinated sacrificial bond networks [17] and atomic-level understanding of the AlCl_3_/ZnCl_2_ dual-salt dissolution mechanism [18] also provide new routes to gel toughening and cellulose valorization. However, most of these works target a single cellulose source or a single performance goal; how the structural characteristics of different fiber raw materials systematically affect the overall performance of the resulting gels still lacks a direct comparison.

Introducing high concentrations of inorganic salts into hydrogels is one of the effective strategies for addressing the above problems [14,15]. High concentrations of salt ions can lower the chemical potential of free water through hydration, significantly depress the freezing point of the system, and inhibit ice-crystal nucleation and growth, endowing hydrogels with excellent antifreezing performance [11,19]. According to colligative properties, the higher the concentration of solute particles in solution, the greater the freezing-point depression. Multivalent metal salt ions (e.g., Zn^2+^, Al^3+^) dissociate into multiple ions in water, further enhancing this effect [17]. Meanwhile, Zn^2+^ can penetrate into cellulose and disrupt its intramolecular and intermolecular hydrogen bond networks, enabling effective dissolution and regeneration of cellulose [20]. The dissociated ions also serve as charge carriers that impart ionic conductivity to the system. In addition, dynamic coordination bonds formed between metal ions and functional groups on polymer chains can act as sacrificial bonds that preferentially break under stress to dissipate external energy, improving the toughness and cyclic stability of hydrogels [17,21]. Such dynamic coordination bonds are reversible—they can re-form after breaking, allowing the hydrogel to recover its network integrity after large deformation, which is an effective route to fatigue resistance [21]. Among various metal ions, Zn^2+^ has attracted considerable attention because of its moderate coordination ability and versatile coordination numbers, forming reversible coordination bonds with hydroxyl (-OH) and amide (-CONH_2_) groups on polymer chains [17]. Al^3+^, with its high charge density (+3) and extremely small hydration radius (ca. 0.535 Å), can effectively penetrate and disrupt the hydrogen bond network between cellulose chains, enabling rapid cellulose dissolution [18]. In this work, an AlCl_3_/ZnCl_2_ dual metal–salt system is employed to construct high-performance conductive hydrogels through the division of labor between Al^3+^ and Zn^2+^: Al^3+^ acts as a hydrogen bond dissociation agent that disrupts interchain hydrogen bonds of cellulose to facilitate Zn^2+^ penetration, whereas Zn^2+^ serves as a stabilizer and crosslinker that coordinates with hydroxyl groups to prevent chain re-aggregation and forms a dynamic crosslinked network during polymerization. Zn^2+^ simultaneously functions as the conductive charge carrier, while Al^3+^ also participates in network construction; the complementary roles of the two metal salts jointly improve the overall mechanical and electrochemical performance of the material.

Cellulose, the most abundant renewable polymer in nature, possesses hydroxyl-rich molecular chains that can form physical crosslinks with polyacrylamide (PAM) network via hydrogen bonding, constructing a rigid–flexible double-network structure [22,23]. In this double-network structure, the rigid cellulose network builds a dense skeleton through physical crosslinks such as hydrogen bonds, which dissipate large amounts of external stress energy through sacrificial bond rupture upon loading [24,25]; the flexible PAM network is maintained by covalent crosslinks and retains resilience under large deformation, preventing irreversible damage [22,25]. The combination of the two networks improves the mechanical properties of the hydrogel. Cellulose-based gels can also directly serve as solid electrolytes that trap and conduct Zn^2+^, showing application potential in flexible supercapacitors and related devices [26]. Moreover, the hydroxyl groups on cellulose chains bind part of the water molecules through hydrogen bonding, which complements with the hydration effect of metal ions to enhance the antifreezing capability of the system [27,28]. The contribution of cellulose to antifreezing performance mainly operates in two dimensions [29,30]: on the one hand, its hydroxyl groups convert “free water” into “bound water” through hydrogen bonding, reducing the number of freezable water molecules; on the other hand, the cellulose network acts as a physical skeleton that suppresses excessive ice-crystal growth during freezing and thawing, safeguarding the integrity of the network structure. It is worth noting that the degree of polymerization (DP) of cellulose varies significantly among different plant sources—cotton cellulose can reach a DP of 1000–3000, whereas bagasse cellulose usually has a lower DP. The DP level directly determines the physical entanglement density between cellulose chains and the PAM network as well as the number of hydrogen bonding sites, thereby affecting the network compactness, ion channel connectivity and antifreezing stability of the gel [24,31,32]. High-DP cellulose (e.g., cotton) has long molecular chains and abundant entanglement points, offering excellent theoretical reinforcement, but excessively high DP may increase dissolution difficulty and impair dispersion uniformity; low-DP cellulose (e.g., bagasse) dissolves readily but provides relatively limited reinforcement [32]. Therefore, selecting fiber raw materials according to performance requirements is of great importance. However, existing studies on antifreezing conductive cellulose/PAM double-network hydrogels based on the AlCl_3_/ZnCl_2_ system are mostly confined to a single cellulose source, and the regulation mechanism of DP differences among different fiber sources on the mechanical, electrical and antifreezing properties of the gels remains insufficiently explored [23,25].

Based on the above research gap, this work employs an AlCl_3_/ZnCl_2_ aqueous solution as the solvent system and uses four commercial bleached pulp boards (eucalyptus, pine, bagasse and cotton fibers) as cellulose raw materials. After dissolution, acrylamide is polymerized in situ to construct a series of cellulose/PAM double-network conductive hydrogels. Through six groups of stepwise control experiments (pure PAM, ZnCl_2_–PAM and four fiber-reinforced composite gels), the effects of fiber raw material source on the microstructure, mechanical properties (compression and after freeze–thaw cycling) and electrochemical properties (electrochemical impedance spectroscopy (EIS) and strain-sensing response) of the gels are systematically investigated, aiming to establish structure–property correlations between the structural characteristics of the cellulose raw materials (DP, crystallinity, morphology and dissolution–regeneration state) and the performance of the double-network gels and to propose a mechanistic framework for selecting fiber raw materials for antifreezing conductive hydrogels.

## 2. Results and Discussion

### 2.1. Characterization of Cellulose Raw Materials

#### 2.1.1. Fiber Morphology

Figure 1a–d shows scanning electron microscopy (SEM) images of the four pulp fibers. Eucalyptus fibers (E) are mostly flat ribbons with relatively smooth surfaces, showing twists and kink nodes, with an apparent width of ca. 15–30 μm. Pine fibers (P) are thick, stiff and straight, with an apparent width of ca. 25–40 μm, rough surfaces and pronounced external fibrillation, with fine fibrils peeling off from the fiber surface. Bagasse fibers (S) show the most heterogeneous morphology, in which slender fibers (ca. 10–20 μm) coexist with wide, flake-like parenchyma cell debris, with abundant fine fiber fractions and surface debris. Cotton fibers (C) are uniform in thickness, with an apparent width of ca. 15–25 μm, appearing as smooth-surfaced ribbons with the natural convolutions characteristic of cotton and the fewest surface defects. The differences among the four fibers in width, surface roughness and degree of fibrillation will directly affect their swelling and dissolution behavior in the AlCl_3_/ZnCl_2_ system, and consequently determine the reinforcement effectiveness in the gel network.

#### 2.1.2. FT-IR Analysis

Figure 1e shows the Fourier-transform infrared (FT-IR) spectra of the four pulp boards. The four spectra are highly consistent in peak position and shape, all presenting the typical absorptions of cellulose: the broad band near 3330 cm^−1^ is assigned to hydrogen bonded O–H stretching, 2897 cm^−1^ to C–H stretching, 1642 cm^−1^ to H–O–H bending of adsorbed water, 1428, 1366 and 1316 cm^−1^ to CH_2_ bending, C–H bending and CH_2_ wagging, respectively, 1160 cm^−1^ to antisymmetric C–O–C glycosidic stretching, 1105, 1052 and 1029 cm^−1^ to C–O stretching at different sites, and 896 cm^−1^ to the characteristic absorption of the β-1,4-glycosidic bond [33]. The deviations of corresponding peak positions among the four spectra do not exceed 5 cm^−1^, indicating that the chemical structures of the four raw materials are essentially identical. No C=O absorptions of hemicellulose acetyl groups and uronic esters near 1730 cm^−1^, nor lignin aromatic-ring characteristic peaks at 1590, 1505 and 1240 cm^−1^, are observed, confirming that all four pulp boards are fully bleached with high cellulose purity. The lateral order index (LOI, definition and calculation in Section 4.3) derived from the absorbance ratio of the crystalline band to the amorphous band [33] ranks as cotton (0.88) > pine (0.58) > eucalyptus (0.38) > bagasse (0.29), qualitatively indicating the highest crystalline order of cotton cellulose.

**Figure 1:**
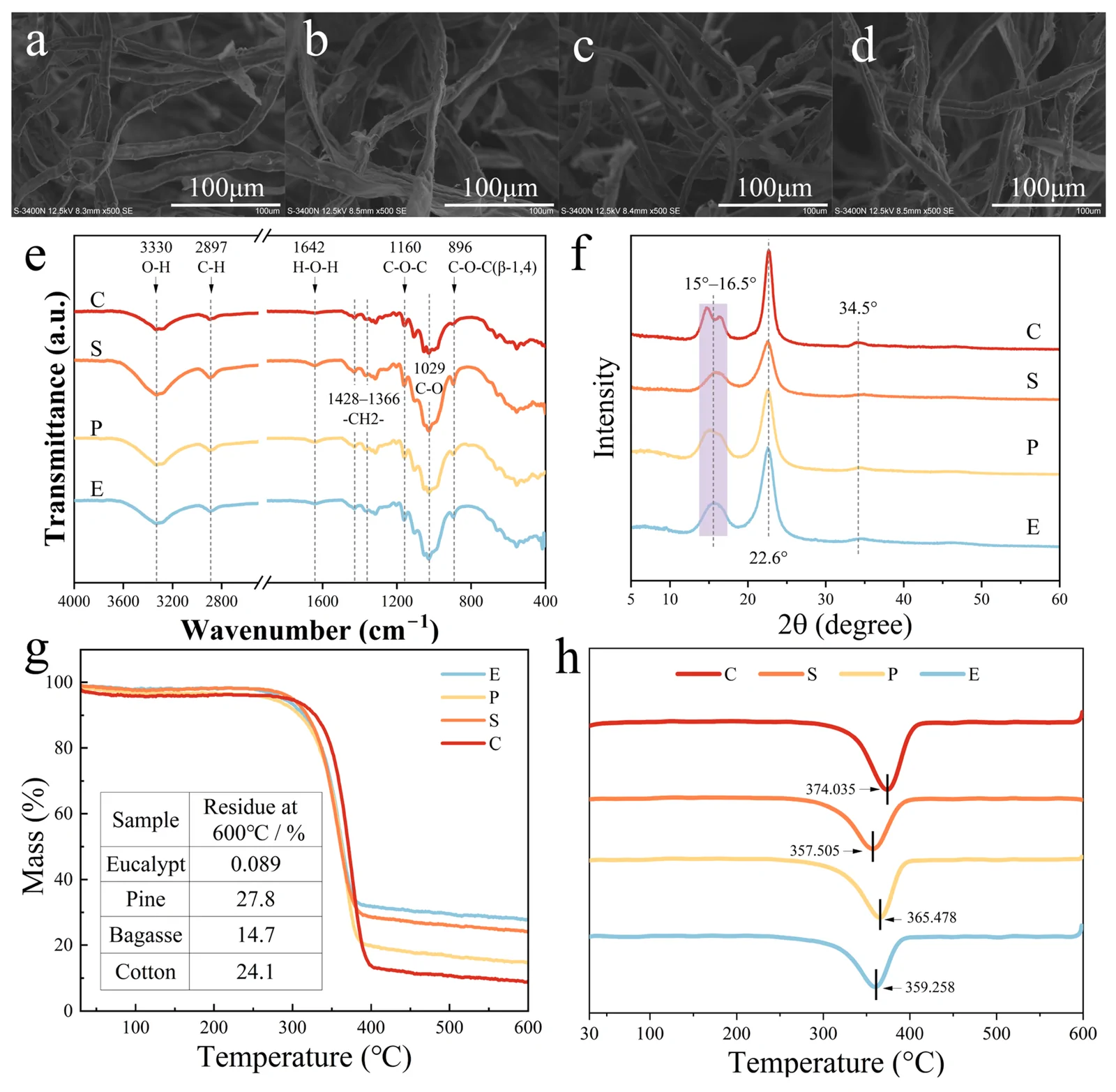
Characterizations of the four plant-fiber raw materials. (**a**–**d**) SEM images of eucalyptus (E), pine (P), bagasse (S) and cotton (C) pulp fibers; (**e**) FT-IR spectra, (**f**) XRD patterns, (**g**) TG and (**h**) DTG curves of the four pulp boards.

#### 2.1.3. Thermal Stability

Figure 1g,h shows the thermogravimetric (TG) and derivative thermogravimetric (DTG) curves of the four pulp boards. All four pulps exhibit consistent thermal degradation processes: the slight weight loss before 100 °C (2.2–4.0%) corresponds to the removal of adsorbed water; the main degradation stage occurs at 300–390 °C, with a single DTG peak corresponding to glycosidic bond scission and depolymerization of cellulose, consistent with the typical thermal behavior of bleached chemical pulps [34]. Resolvable differences in thermal stability exist among the four pulps. Compared with the temperature at 10% weight loss, the order is cotton (330 °C) > bagasse (320 °C) > eucalyptus (316 °C) > pine (308 °C); the DTG peak temperatures follow the same order: cotton 374 °C, pine 365 °C, eucalyptus 360 °C and bagasse 358 °C. The main degradation peak of cotton pulp is 16 °C higher than that of bagasse pulp, indicating longer cellulose chains and more perfect crystalline regions, consistent with the rankings of DP and crystallinity. The char residue at 600 °C ranks in the opposite order: eucalyptus 27.8%, bagasse 24.1%, pine 14.7% and cotton 8.9%. The lowest char residue of cotton pulp reflects its high α-cellulose content and low ash and char-forming impurities, whereas the higher char residues of eucalyptus and bagasse pulps are associated with their higher inorganic ash contents.

#### 2.1.4. X-Ray Diffraction and Crystallinity

Figure 1f shows the X-ray diffraction (XRD) patterns of the four pulp boards. All four raw materials exhibit typical diffraction features of native cellulose I: the overlapped diffraction peaks of the (1–10) and (110) crystal planes at 2θ ≈ 15–16.5°, the strong diffraction peak of the (002) plane at ca. 22.5°, and the weak diffraction peak of the (004) plane at ca. 34.5° [35]. The relative crystallinity index (CrI) was estimated using the Segal empirical formula [36], and the results are listed in Table 1: cotton (88.2%) > eucalyptus (79.1%) > pine (78.6%) > bagasse (75.2%). Cotton cellulose has the highest crystallinity, with the clearest splitting of its (1-10) and (110) peaks (discernible doublet at 14.8° and 16.4°), corroborating its highest DP (2327) and thermal stability; eucalyptus and pine show close crystallinities; bagasse is the lowest, consistent with its heterogeneous morphology, abundant parenchyma cell debris, and lowest DP (914). The Segal method is an empirical relative index whose systematically overestimated values are an intrinsic feature of the method; it is used here only for relative comparison among the four raw materials.

#### 2.1.5. Degree of Polymerization

The DP values (Table 1) follow the order cotton (2327) > pine (1356) > eucalyptus (1082) > bagasse (914), consistent with the trends in CrI, LOI and thermal stability. DP reflects molecular chain length, whereas CrI and LOI reflect crystalline packing perfection; these parameters are not necessarily synchronized (e.g., pine and eucalyptus exhibit similar CrI but differ in LOI and DP). Although a higher DP theoretically provides more entanglement and hydrogen bonding sites for network reinforcement [32], it simultaneously increases dissolution difficulty and may impair dispersion uniformity. Consequently, DP alone does not dictate gel performance: the pine-based gel, despite ranking second in DP, shows the best low-temperature and freeze–thaw durability (Section 2.3 and Section 2.5), underscoring that the dissolution–regeneration state and interfacial bonding quality are equally decisive.

### 2.2. Microstructure and Chemical Characteristics of the Hydrogels

#### 2.2.1. FT-IR Spectra and Hydrogen Bonding/Coordination Interactions

Pure PAM exhibits a doublet of N–H antisymmetric and symmetric stretching vibrations at 3330 and 3182 cm^−1^; 1645 and 1601 cm^−1^ correspond to the amide I band (C=O stretching) and amide II band (N–H bending), respectively (Table 2); 2930 and 1410 cm^−1^ are assigned to main chain C–H stretching and CH_2_ bending, consistent with the formation of a saturated PAM main chain. These FT-IR signals alone cannot quantitatively prove complete monomer conversion or exclude residual monomers/oligomers (such quantification would require NMR or chromatographic analysis). After introducing AlCl_3_/ZnCl_2_, the amide II bands of the five salt-containing gels uniformly red-shift to 1581–1583 cm^−1^, and the N–H symmetric stretching band blue-shifts from 3182 cm^−1^ to 3198–3208 cm^−1^, whereas the amide I band remains essentially unchanged (1644 cm^−1^). The absence of a resolvable amide-I shift indicates that strong coordination between the carbonyl oxygen and metal ions is not spectrally evident under our measurement conditions (weaker or heterogeneous interactions cannot be excluded), whereas the shifts in the amide II and N–H bands indicate that Zn^2+^/Al^3+^ mainly perturb the hydrogen bonding association of the amide groups. Therefore, we attribute the toughening mechanism to the combined action of hydrogen bond reorganization, chain entanglement and pore wall architecture rather than to strong metal–carbonyl coordination. The absorption peak at 757 cm^−1^ appears only in the salt-containing gels and is attributed to the metal–ion coordination environment and bound water. All four fiber-modified gels show a cellulose C–O skeletal vibration peak at 984–986 cm^−1^, which is absent in pure PAM and Zn-PAM, indicating that the cellulose skeletal structure remains detectable in the network after processing in the network after dissolution–regeneration. Although the cellulose crystalline diffraction of Zn-PAM/E has disappeared in XRD (Figure 2b), its 984 cm^−1^ skeletal absorption remains discernible, consistent with extensive dissolution–regeneration of the eucalyptus cellulose. However, FT-IR and XRD cannot assess molecular-weight changes, and partial chain scission or DP reduction during processing cannot be excluded. The overall FT-IR features of the four fiber-modified gels are similar. Considering the spectral resolution (4 cm^−1^) and the uncertainty in peak position determination, differences of 1–3 cm^−1^ fall within the experimental error and are therefore not interpreted mechanistically in this work. Dynamic coordination between metal ions and polar groups has been proven to be an effective route to improving the energy dissipation and ion transport capability of gel networks [21].

**Table 2:** Peak assignments for FT-IR spectra.

Wavenumbers/cm^−1^	Assignments
3330	O–H (cellulose)/N–H (PAM) stretching (overlap)
~3200	N–H symmetric stretching
1644	Amide I (C=O stretching)
1601/1582	Amide II (N–H bending)
1410	CH_2_ bending
1219	C–N stretching/skeletal vibration
1112	C–N stretching + C–O–C (cellulose) overlap
985	C–O skeletal vibration of cellulose
757	Zn^2+^/Al^3+^ coordination environment/bound water

#### 2.2.2. X-Ray Diffraction and the Aggregation State of Regenerated Cellulose

Figure 2b shows the XRD patterns of the six freeze-dried gels. Pure PAM presents only a broadened amorphous halo at 2θ ≈ 21°, consistent with its amorphous structure. After introducing AlCl_3_/ZnCl_2_, the halo shape of Zn-PAM changes (the centroid shifts toward 25–30°), reflecting the influence of the high-ionic-strength environment on the short-range aggregation of PAM segments, but no sharp crystalline diffraction peaks appear. Notably, the characteristic cellulose I diffractions (the 15–16.5° doublet and the 22.5° main peak) completely disappear in all four fiber-modified gels, indicating that no long-range cellulose I crystalline domains were detected in the four fiber-modified gels within the detection limit of the measurement, i.e., the original crystalline order was largely destroyed—ZnCl_2_ hydrates possess ionic-liquid-like characteristics, and hydrated Zn^2+^/Cl^−^ can efficiently disrupt the intra- and intermolecular hydrogen bond networks of cellulose [37]. Meanwhile, no long-range cellulose II crystalline diffraction appears in the patterns, indicating that no large-scale cellulose I → II recrystallization occurred during dissolution–regeneration and in situ polymerization, and the regenerated cellulose exists in the PAM matrix mainly in an amorphous or near-molecularly dispersed state, consistent with the clearly discernible cellulose skeletal absorptions at 984–986 cm^−1^ in the four gels: the cellulose skeleton is chemically intact but exists in a disordered condensed state. Compared with the blank Zn-PAM pattern (data processing described in Section 4.3), Zn-PAM/C, Zn-PAM/S, and Zn-PAM/P exhibit weak excess broadened scattering in the 19–22° region, which can be assigned to a small amount of cellulose II short-range order in the regenerated cellulose [35], with an intensity order of cotton > bagasse > pine, all at very low levels. The halo shape of Zn-PAM/E differs markedly from the others (the center shifts to ca. 16.5°), suggesting that its regenerated cellulose forms a different short-range aggregated structure with PAM/salt (this difference may also be related to the compactness of the freeze-dried sample). The above results indicate that, in this system, the differences among fiber raw materials are not manifested in the amount of crystalline retention, but mainly in the dispersion/aggregation state of regenerated cellulose in the matrix and the pore wall architecture constructed, which is the structural origin of the divergence in mechanical and electrochemical performance.

#### 2.2.3. Cross-Sectional Morphology and Pore Structure

Figure 2c–h shows SEM images of the freeze-dried cross-sections of the six hydrogels. Pure PAM exhibits an irregular, torn pore structure with a broad pore-size distribution (ca. 5–25 μm), and its pore walls are thin and mostly curled flakes. Blank Zn-PAM shows highly regular polygonal honeycomb pores (ca. 10–20 μm) with thin, smooth and well-defined walls—the most uniform structure among the six groups—indicating that the high-ionic-strength environment promotes uniform phase separation and crosslinking of the PAM network. The cross-sectional morphologies of the four fiber-modified gels differ significantly: Zn-PAM/E has fine, dense pores (ca. 2–8 μm) with thin and brittle walls and the most compact structure, in which the eucalyptus cellulose, after complete amorphization, is dispersed at a near-molecular level in the matrix without forming an independent skeleton; Zn-PAM/P has rough pore walls covered with regenerated microfibrils, forming a hierarchical pore structure (pore cells of ca. 5–20 μm with sub-micron fibril networks embedded in the walls); Zn-PAM/S has pore cells of ca. 10–30 μm with thick walls (ca. 5–10 μm) containing secondary micropores, indicating enrichment and aggregation of regenerated cellulose in the pore walls; Zn-PAM/C consists of continuous, smooth membranous pore walls enclosing open large pore cells (ca. 15–40 μm), with interconnecting windows on the membranes, showing the best pore openness among the six groups. The cross-sectional morphology corresponds well with the crystal structure and macroscopic performance: the pore wall thickness ranks bagasse > pine > cotton > eucalyptus; the open membranous channels of Zn-PAM/C correspond to its highest room temperature conductivity; and the fine, thin-walled structure of Zn-PAM/E is associated with its “stiffening but toughness-reduced” tensile behavior and its relatively large stiffening amplitude at −30 °C.

### 2.3. Mechanical Properties

#### 2.3.1. Tensile Properties at Room Temperature

Figure 3a shows the stress–strain curves of the six hydrogels under uniaxial tension at room temperature. All samples exhibit typical features of soft hydrogels: near-linear elasticity in the low-strain region, followed by stable load-bearing elongation until fracture, without an obvious yield plateau. The tensile strength, elongation at break and elastic modulus of pure PAM are 71.51 kPa, 1346% and 26.7 kPa, respectively. After introducing AlCl_3_/ZnCl_2_, the tensile strength of Zn-PAM increases to 104.08 kPa (+45.5%) and the elastic modulus to 48.6 kPa (+82.0%), but the elongation at break decreases to 937% (−30.4%): the coordination between Zn^2+^/Al^3+^ and amide groups, together with the high-ionic-strength environment, increases the effective crosslinking density of the network, making the gel stiffer but less stretchable, while its toughness (605.9 kJ·m^−3^) remains essentially the same as that of pure PAM (594.0 kJ·m^−3^).

After modification with the four plant fibers, the tensile properties of the gels diverge markedly (Figure 3b,c). Zn-PAM/C shows the best comprehensive performance: a tensile strength of 105.25 kPa, elongation at break of 1566% and toughness of 910.5 kJ·m^−3^; while maintaining the strength level of Zn-PAM, its elongation at break increases by 67.0% and its toughness by 50.3% relative to Zn-PAM, achieving simultaneous strengthening and toughening [38]. Zn-PAM/E has a strength of 93.26 kPa and an elastic modulus of 40.2 kPa, the second-highest stiffness after Zn-PAM, but its elongation at break drops to 980%, showing a “stiffening but toughness-reduced” character. Zn-PAM/S, with a strength of 77.56 kPa and an elongation at break of 1359%, behaves as “soft yet stretchable”, with a toughness (593.0 kJ·m^−3^) comparable to pure PAM. Zn-PAM/P shows the lowest values in all indices (56.23 kPa, 843%, 303.4 kJ·m^−3^), decreasing by 21.4% and 48.9% in strength and toughness relative to pure PAM, respectively.

The above divergence corresponds to the raw material structural differences in Section 2.1 and Section 2.2: cotton cellulose has the highest DP (2327), high crystallinity and uniform fiber morphology; the continuous regenerated-cellulose phase formed after dissolution–regeneration cooperates with the matrix in load bearing and effectively hinders crack propagation. Eucalyptus cellulose is the most uniformly dispersed after dissolution–regeneration, and the regenerated cellulose phase raises the modulus as a rigid filler, but the lack of a continuous skeleton leads to reduced elongation at break. Notably, although pine pulp has the second-highest DP (1356), its gel shows the lowest tensile performance, indicating that DP is not the sole factor determining the reinforcement effect: pine fibers are thick and heavily fibrillated on the surface, their dissolution–regeneration in the AlCl_3_/ZnCl_2_ system is nonuniform, and the rough microfibrillar walls formed after regeneration bond weakly with the PAM matrix, resulting in low load-transfer efficiency. This demonstrates that the dissolution–regeneration state of cellulose and the interfacial bonding quality jointly determine the final reinforcement effect together with the raw material DP.

#### 2.3.2. Compression and Cyclic Stability at Room Temperature

Figure 4a shows the single compression stress–strain curves of the six gels at 70% strain at room temperature. All curves rise smoothly without fracture inflection points, and the samples remain intact at 70% large strain. The compression behavior follows a different ranking from tension (Figure 4c): Zn-PAM exhibits the highest stress at 70% strain (σ_70_ = 554.07 kPa) and compressive modulus (214.7 kPa), markedly higher than the other groups; the σ_70_ values of the four fiber-modified gels rank as Zn-PAM/E (388.45 kPa) > Zn-PAM/C (366.69 kPa) > Zn-PAM/P (300.22 kPa) > Zn-PAM/S (243.51 kPa), with compressive moduli of 143.9, 126.0, 107.9 and 78.3 kPa, respectively. Comparison with the tensile properties shows that compression resistance is dominated by the densely ion-crosslinked PAM matrix in the high-ionic-strength environment; the introduction of the fiber phase dilutes and perturbs the ion-crosslinked network of the matrix to some extent, lowering the compressive modulus and σ_70_ relative to Zn-PAM, whereas under tensile loading the load-bearing, orientation and crack-deflection effects of the fiber network are more pronounced, so that Zn-PAM/C and Zn-PAM/E exhibit obvious reinforcement in tension. Figure 4b shows the normalized stress retention curves over 20 compression cycles at 70% strain at room temperature. After 20 cycles, the stress retentions are 103.1% (PAM), 99.0% (Zn-PAM), 116.1% (Zn-PAM/E), 100.6% (Zn-PAM/P), 99.6% (Zn-PAM/S) and 111.3% (Zn-PAM/C), with the minimum value during the whole cycling no lower than 93.9% (Zn-PAM/S, 4th cycle); none of the six groups shows fatigue decay. Zn-PAM and Zn-PAM/S show a slight initial stress drop (ca. 4–6%) followed by gradual recovery, reflecting the self-adaptive equilibrium of dynamic hydrogen bond dissociation–reconstruction during compression cycling; the stresses of Zn-PAM/E and Zn-PAM/C increase continuously, rising by 16.1% and 11.3% after 20 cycles, respectively, showing a “mechanical training” effect [39]—cyclic compression promotes the rearrangement of cellulose–matrix interfacial hydrogen bonds and the oriented densification of the network, so that the effective crosslinking density increases rather than decreases. The excellent cyclic stability of the six gels originates from the synergy between the covalently crosslinked PAM skeleton and reversible physical crosslinks (cellulose–amide hydrogen bonds and Zn^2+^/Al^3+^ coordination): the rigid covalent network ensures structural integrity under large strain, while dynamic sacrificial bonds repeatedly break and re-form during cycling to dissipate energy [40].

#### 2.3.3. Low-Temperature Compression Performance

Figure 4d shows the single compression stress–strain curves of the five gels at −30 °C (pure PAM is not included because its stress exceeded the load cell limit after freezing). All five samples remain intact at 70% strain, with curves rising smoothly and no embrittlement inflection points—none of them undergo freeze-induced hardening: the σ_70_ values are 627.7 kPa (Zn-PAM), 524.2 kPa (Zn-PAM/E), 358.0 kPa (Zn-PAM/P), 296.1 kPa (Zn-PAM/S) and 466.3 kPa (Zn-PAM/C), only 1.13–1.35 times those at room temperature (Figure 4a), and the compression resistance ranking is the same as at room temperature (Zn-PAM > Zn-PAM/E > Zn-PAM/C > Zn-PAM/P > Zn-PAM/S). The moderate increase in modulus originates from the crystallization of part of the freezable water and the matrix stiffening caused by restricted segmental motion; the hydration-based antifreezing effect of high-concentration Zn^2+^/Al^3+^ and the water-binding capability of the cellulose network jointly guarantee the compressibility of the gels at −30 °C. Among them, Zn-PAM/E shows the largest stiffening amplitude (×1.35), which is related to the relatively high proportion of freezable water in its dense, thin-walled pores, but this is far from freeze-induced failure. Figure 4e shows the normalized stress retention curves over 20 compression cycles at 70% strain at −30 °C (pure PAM, which exceeded the load cell limit under this condition, is not included). After 20 cycles, the stress retentions of Zn-PAM, Zn-PAM/E, Zn-PAM/P, Zn-PAM/S and Zn-PAM/C are 96.4%, 110.9%, 96.4%, 112.8% and 110.8%, respectively. None of the five samples suffers fatigue failure during low-temperature cycling: the stresses of Zn-PAM/E, Zn-PAM/S and Zn-PAM/C increase rather than decrease (+10.9%, +12.8% and +10.8%, respectively), indicating that the “mechanical training” effect remains significant at −30 °C [39]—the cellulose physical network and the PAM network become regularly oriented and locally densified under repeated compression, so that the load-bearing capacity is enhanced instead; Zn-PAM and Zn-PAM/P show slight declines (ca. −3.6%) that stabilize in the middle-to-late cycling stage, corresponding to a transient lag in dynamic hydrogen bond dissociation–reconstruction. Compared with the room temperature cycling results (Figure 4b), the retentions of all groups at low temperature are overall comparable, indicating that the gel networks retain good elastic recovery and dynamic-bond reorganization activity at −30 °C, further confirming the low-temperature service reliability of this system [41].

### 2.4. Mechanical Properties After Freeze–Thaw Cycles

To evaluate the structural stability of the conductive gels after repeated low-temperature service, the six hydrogels were sealed and subjected to five freeze–thaw cycles at −40 °C (each cycle: freezing for 8 h followed by sealed thawing at room temperature for 2 h), and uniaxial tensile tests were performed after they recovered to room temperature. The results are shown in Figure 5.

Figure 5a shows the stress–strain curves of the gels after freeze–thaw. Comparison with the pre-freeze–thaw curves (Figure 3a) reveals markedly divergent responses of the six samples to freeze–thaw cycling (Figure 5b): the tensile strength of pure PAM increases from 71.51 kPa to 99.46 kPa (retention 139.1%), its elastic modulus rises from 26.7 kPa to 38.3 kPa, while its elongation at break slightly decreases from 1346% to 1258% (retention 93.4%). The strength of Zn-PAM is essentially unchanged (101.58 kPa, retention 97.6%), but its elongation at break decreases to 600% (retention 64.0%) and its toughness decreases by 38.6%. Among the four fiber-modified gels, Zn-PAM/E, Zn-PAM/P and Zn-PAM/S are strengthened rather than weakened after freeze–thaw: their strength retentions are 121.6%, 146.1% and 152.2%, and their elongation retentions are 113.6%, 150.4% and 131.0%, respectively; in particular, Zn-PAM/S reaches a tensile strength of 118.03 kPa, an elongation at break of 1780% and a toughness of 992 kJ·m^−3^ after freeze–thaw, the highest of all six groups in all three indices. In contrast, Zn-PAM/C, which performs best in the pristine state, deteriorates most severely after freeze–thaw, with strength and elongation retentions of only 62.6% and 44.6%, and its toughness dropping to 308 kJ·m^−3^ (retention 33.8%).

The above divergence can be attributed to the competition between two opposite mechanisms during freeze–thaw. On the one hand, at −40 °C the freezable water in the gel partially crystallizes; ice-crystal growth expels solutes and polymer chains into unfrozen salt-rich microdomains, where cellulose and PAM chains are locally concentrated and compressed, forming new hydrogen bonds and physical entanglement points at the cellulose–matrix interface upon thawing—namely, “freeze–thaw-induced physical cross-linking” [42,43]—which permanently strengthens the network. On the other hand, ice-crystal growth may also pierce the network and disrupt the interfacial bonding between cellulose and the matrix, causing irreversible damage. For Zn-PAM/P and Zn-PAM/S, whose pristine networks are still imperfect and have considerable room for improvement, the former mechanism dominates, and freeze–thaw acts as a physical post-crosslinking treatment that greatly enhances performance. Zn-PAM/E shows the largest stiffening amplitude during in situ compression at −30 °C, yet its performance increases rather than decreases after thawing (retention 121.6%), indicating that its low-temperature structural changes are essentially reversible after thawing. The pristine network of Zn-PAM/C is highly uniform with well-developed interfacial bonding, so ice-crystal growth instead disrupts this uniform structure, resulting in marked deterioration after freeze–thaw. It should be emphasized that the above data reflect the net effect after five freeze–thaw cycles; since no intermediate characterization was performed after each cycle, we cannot distinguish whether the structural evolution is progressive, stepwise, or dominated by the initial cycles. The proposed mechanism—competition between freeze-concentration-induced physical crosslinking and ice-crystal-induced interfacial damage—is inferred from the mechanical results and the literature [42,43], and direct structural evidence (e.g., post-freeze–thaw FT-IR/XRD) is not yet available.

### 2.5. Electrochemical and Strain-Sensing Properties

To investigate the influence of plant-fiber species on the ion transport performance and low-temperature adaptability of the conductive hydrogels, the blank group (Zn-PAM) and the composite hydrogels modified with eucalyptus (Zn-PAM/E), pine (Zn-PAM/P), bagasse (Zn-PAM/S), and cotton (Zn-PAM/C) fibers were tested. The results are shown in Figure 6a–g and Table 3.

#### 2.5.1. AC Impedance Spectra and Ionic Conductivity

Figure 6a–d shows the Nyquist plots of Zn-PAM and the four fiber-modified hydrogels at 20, 0, −20 and −40 °C. The curves exhibit the characteristic pattern of a high-frequency intercept followed by a low-frequency sloping line, typical of blocking electrode/ionic conductor behavior; the bulk resistance was obtained from the high-frequency intercept and converted into ionic conductivity, as shown in Figure 6e. At 20 °C, the conductivity ranks as Zn-PAM/C (50.53 mS·cm^−1^) > Zn-PAM/S (44.41) > Zn-PAM (38.79) > Zn-PAM/E (23.20) > Zn-PAM/P (16.29). This difference is consistent with the SEM morphology (Figure 2e–h): cotton cellulose regenerates into continuous membranous pore walls with open and spacious channels, and bagasse-regenerated cellulose forms thick walls with uniform pores, both offering low ion-migration resistance; eucalyptus-regenerated cellulose is overly finely dispersed and forms only fine, thin-walled pores, while pine regenerated microfibrils roughen and constrict the pore walls, giving narrow effective pore sizes and high migration resistance, and accordingly lower room temperature conductivities. The ZnCl_2_ loading in this work is 27.24 g per 100 g of gel (ca. 2 mol·L^−1^), only about one quarter of that in “water-in-salt” systems [44], yet the room temperature conductivity is of the same order of magnitude—Fu et al. [45] reported a polyzwitterionic conductive hydrogel filled with 7.5 mol/L ZnCl_2_ showing a conductivity of 74.3 mS·cm^−1^ at 25 °C; with a low salt loading, Zn-PAM/C in this work reaches 68% of that level, indicating that the promotion of ion migration by regular cellulose channels can partially substitute for high salt loading.

As the temperature decreases, the conductivities of all samples decline, but the attenuation amplitudes differ significantly. At 0 °C, all five salt-containing gels retain over 40% of their room temperature conductivity, with Zn-PAM/C showing the lowest retention (41.4%); at −20 °C, Zn-PAM/S ranks first with 7.30 mS·cm^−1^; at −40 °C, Zn-PAM/P still maintains 0.83 mS·cm^−1^ with a room temperature retention of 5.1%, both being the best among the five groups—approximately 3.5 and 5 times those of the eucalyptus gel (0.24 mS·cm^−1^, 1.0%), and 28 and 51 times those of the cotton gel (0.03 mS·cm^−1^, 0.1%), respectively. Zn-PAM/C, the best room temperature conductor, attenuates most drastically at −40 °C, whereas Zn-PAM/P, the worst at room temperature, shows the strongest low-temperature retention capability, indicating that room temperature and low-temperature conduction are dominated by different structural factors. The anodic electrochemical stability of the five salt-containing gels was further evaluated by linear sweep voltammetry (LSV), exhibiting stability limits of 1.23–1.34 V (Figure S3 and Table S3), which confirms their suitability as gel electrolytes for low-voltage flexible electronics.

#### 2.5.2. Antifreezing and Conductive Mechanism

The low-temperature conductivity of this system originates from the synergy between “concentrated-salt antifreezing” and “cellulose-confined water retention” [46]. Zhang et al. [47] lowered the solid–liquid transition temperature of the electrolyte to −114 °C by tuning the ZnCl_2_ concentration to 7.5 m; the essence is that high concentrations of Zn^2+^/Cl^−^ disrupt the hydrogen bond network among water molecules, existing as solvated ions such as [Zn(H_2_O)_2_Cl_4_]^2−^, [ZnCl]^+^ and [Zn(H_2_O)_6_]^2+^, thereby suppressing ice-crystal nucleation. The “water-in-salt” polyzwitterionic conductive gel of Fu et al. [45] exhibits a conductivity decreasing from 74.3 to 15.6 mS·cm^−1^ over the wide temperature range of +25 to −60 °C, and the assembled zinc-ion hybrid capacitor retains 84.6% of its capacity after 100,000 cycles at −40 °C, further confirming the decisive role of the highly concentrated ZnCl_2_ solvation structure in low-temperature ion transport. The cellulose-nanofiber-based ionic gels reported by Han et al., which combine high conductivity with healability at low temperatures, likewise confirm the effectiveness of this strategy in biomass gel systems [29]. The inconsistency between the room temperature and low-temperature conductivity rankings in this work can be explained by a dual-factor framework of “pore-channel structure” and “bound water”. At room temperature, the ion transport resistance mainly comes from channel tortuosity [16]: the regular channels of cotton and bagasse gels afford higher room temperature conductivities, whereas the channel defects of eucalyptus and pine gels lead to lower conductivities. At low temperatures, the key factor shifts to the water-binding capability of the network. Mechanistically, three factors are expected to act together. First, hydrated Zn^2+^/Al^3+^ and Cl^−^ lower the chemical potential and activity of free water, depressing the freezing point of the system. Second, the high ionic strength disrupts the water–water hydrogen bond network, suppressing ice nucleation and growth. Third, the hydroxyl groups of cellulose and the amide groups of PAM bind part of the water molecules, and confinement within the pore walls further inhibits ice-crystal growth; unfrozen salt-rich microdomains can thus serve as local ion transport pathways at subzero temperatures. Pine-regenerated cellulose is retained in the pore walls as microfibrils and forms an efficient dynamic hydrogen bond network with PAM, converting “free water” into “bound water” and reducing the proportion of freezable water; its elastic network maintains channel integrity under ice-crystal expansion stress, so its conductivity and retention at −40 °C both rank first. Although Zn-PAM/C has the most uniform channels, its relatively low bound-water proportion leads to the most drastic low-temperature attenuation. Although the eucalyptus gel has poor continuity of the regenerated-cellulose skeleton, its fully exposed hydroxyl groups can still bind water molecules, so its low-temperature retention (1.0%) is instead higher than those of bagasse (0.2%) and cotton (0.1%). It should be noted that no direct thermal/water-state characterization (e.g., low-temperature DSC, Raman, or NMR) was performed in this work; the above antifreezing mechanism is an inferential interpretation based on the low-temperature mechanical and EIS results combined with the literature, and the ratio of bound to freezable water has not been directly quantified.

To quantify the temperature dependence of ion transport, the conductivities at 20, 0, −20 and −40 °C were fitted to the Arrhenius equation (Figure S2 and Table S2). Over the full measured temperature range, the apparent activation energies are 47.1 (Zn-PAM), 42.5 (Zn-PAM/E), 28.3 (Zn-PAM/P), 52.6 (Zn-PAM/S) and 66.2 kJ·mol^−1^ (Zn-PAM/C), respectively. The ln σ–1000/T curves deviate downward at −40 °C owing to the onset of partial freezing; these values are therefore apparent parameters rather than intrinsic barriers of a single transport mechanism. Zn-PAM/P exhibits the lowest apparent activation energy, i.e., its ion transport is the least temperature-sensitive, which quantitatively supports the view that its microfibril-reinforced pore walls and the associated dynamic hydrogen bond network retain a higher fraction of unfrozen water and maintain continuous ion pathways at subzero temperatures; Zn-PAM/C shows the highest activation energy, consistent with its most drastic conductivity attenuation at −40 °C. Notably, when the fitting is restricted to the non-frozen range (20 to −20 °C), the activation energies of the five gels are very close (19.0–33.1 kJ·mol^−1^, R^2^ ≥ 0.96), indicating that the fiber-source differentiation in low-temperature performance originates mainly from the different freezing-suppression capabilities of the regenerated cellulose networks rather than from intrinsic ion transport barriers above the freezing point.

Compared with existing cellulose-based antifreezing systems, the preparation route of this work has an integration advantage. The double-crosslinked cellulose ionic hydrogel reported by Tong et al. [48] requires post-soaking in NaCl to acquire antifreezing ability, with a minimum melting point of only −22.4 °C and a stretchability of ca. 100% at −24 °C; in this work, the AlCl_3_/ZnCl_2_ aqueous solution simultaneously serves three functions in one system—cellulose solvent, charge-carrier source and antifreezing medium—without any post-treatment, and the measurable EIS temperature range extends to −40 °C. It has been reported that electrolyte design can extend the operating temperature of gel electrolytes to −70 °C [49]; by comparison, the feature of this work lies in the one-pot construction integrating solvent, charge-carrier source and antifreezing medium, and Zn-PAM/P still maintains a usable conductivity of 0.83 mS·cm^−1^ at −40 °C. The κ-carrageenan/PAAm double-network hydrogel designed by Zhou et al. [50] achieves 14.8 mS·cm^−1^ at −40 °C; the cellulose/PAM double-network gel prepared by Li et al. [23] using the AlCl_3_/ZnCl_2_ aqueous system still works normally at −45 °C, demonstrating the excellent antifreezing potential of this dual metal–salt solvent system itself. Compared with Fu et al., there is an order-of-magnitude gap in the −40 °C conductivity retention of this work (5.1% for the best sample), mainly caused by the difference in salt loading, suggesting that moderately increasing the ZnCl_2_ concentration while retaining the fiber skeleton is a direct route to narrowing the low-temperature gap. It should be noted that this work focuses on material-level ion transport, antifreezing and strain-sensing performance; transference-number measurement and, full-device assembly for zinc-based energy storage devices will be carried out in future work.

#### 2.5.3. Strain-Sensing Response and Cyclic Stability

Figure 6f shows the relative resistance change (ΔR/R_0_) of the gels over 0–150% strain. All samples exhibit two-segment linear characteristics: in the 0–50% strain regime, the gauge factor (GF) ranges from 0.49 to 0.76 (R^2^ = 0.82–0.92); above 50% strain, the GF increases to 1.44–1.96 (R^2^ > 0.995). The ΔR/R_0_–strain relationship of ionic conductors is intrinsically quadratic; Song et al. [51] piecewise linearized it in their study on organohydrogel fiber sensing, with GFs of 1.04 and 1.87 in the 0–50% and 50–200% regimes, respectively, basically consistent with the segmentation and sensitivity level of this work. The segmented response originates from a transition of the deformation mechanism: at small strains, elastic deformation of the pore channels dominates; at large strains, PAM-chain orientation and the dissociation–reorganization of cellulose–PAM dynamic hydrogen bonds elongate the Zn^2+^ migration paths, so the resistance response intensifies and tends to become linear.

Different samples present a “sensitivity–stability” trade-off. Blank Zn-PAM has the highest GF (1.96) but lacks a fiber skeleton and has poor mechanical reliability under large deformation; introducing fibers moderately lowers the GF while improving structural stability. Zn-PAM/P has the lowest GF (1.44) but the best linearity in the 0–50% regime (R^2^ = 0.9198), as well as the best mechanical cyclic stability and the most robust conductive pathway; Zn-PAM/S and Zn-PAM/C have intermediate GFs (1.69 and 1.67); the GF of Zn-PAM/E (1.86) is comparable to that of the blank, but its high sensitivity comes at the cost of network fragility. Compared with similar cellulose-based ionic gels, the sensitivity advantage of this work is evident. The cellulose ionic hydrogel reported by Tong et al. [48] shows a GF of 0.297 over 0–220% strain (R^2^ = 0.996); the high-strain-segment GF of this work reaches 1.44–1.96, about 5–7 times that value. The zwitterionic ionic gel of Zheng et al. [52] shows a GF of 0.57 over 0–100% strain, comparable to the low-strain segment of this work (0.49–0.76), but the sensitivity of this work is nearly threefold higher in the high-strain segment, and the sensing material is an all-aqueous system involving neither ionic liquids nor organic-solvent replacement steps. It should be emphasized that the present tests cover only the material-level strain–resistance response and its stability over 20 cycles; sensor-level metrics such as response/recovery time, strain-rate dependence, hysteresis and low-temperature signal-to-noise ratio, as well as long-term environmental/dehydration stability and biocompatibility, have not been evaluated. Therefore, the above results should be regarded as a material-level demonstration of strain-responsive behavior rather than a validated wearable sensor; the bulk ionic conductivity of 0.83 mS·cm^−1^ at −40 °C by itself is also insufficient to guarantee stable low-temperature sensing signals. The integrated design of gel electrolytes with strain-sensing functionality is an important direction for the convergent development of aqueous zinc-based energy storage and wearable electronics [53].

Under 20 cycles of tensile loading at 200% strain (Figure 6g): the response of Zn-PAM rises from 1.80 to 2.05 and then stabilizes (fluctuation ≈ 0% after the 10th cycle); Zn-PAM/P shows a smooth response with the lowest amplitude (1.23 → 1.63), indicating an intact conductive pathway; the fluctuations of Zn-PAM/S (1.58 → 1.81) and Zn-PAM/C (1.58 → 2.00) are 2.7% and 2.8%, respectively; Zn-PAM/E fluctuates the most (3.3%), reflecting the nonuniform rupture and reconstruction of its dense, amorphous network under cyclic deformation. The response amplitudes of all samples gradually increase at the initial stage of cycling (+14% to +29%), reflecting the rearrangement and adaptation of the dynamic hydrogen bond network under repeated deformation.

#### 2.5.4. Summary of Structure–Electrochemical-Performance Correlations

The four fiber-modified gels exhibit clear trade-offs among room temperature conductivity, low-temperature retention, tensile toughness and freeze–thaw stability, so that application-oriented fiber selection is essentially a multi-criteria decision-making (MCDM) problem [54]. Since criterion weights depend on the specific application scenario, we do not provide a single numerical overall ranking here but instead offer criterion-oriented selection guidance: cotton for room temperature-conductivity-priority scenarios, bagasse for strength–conductivity balance, and pine for low-temperature/deformation-adaptation priority. Once application-specific weights are defined, MCDM ranking or rank-aggregation methods [54,55] can be applied to the present data set for a systematic overall ranking.

The regulation of electrochemical performance by the four fibers can be summarized as a triple coupling mechanism of “pore-channel structure–hydrogen bond network–regenerated skeleton”, with different dominant factors under room temperature and low-temperature conditions: room temperature conductivity is determined by channel uniformity—the continuous regenerated membranous pore walls of cotton and the thick regenerated-cellulose walls of bagasse provide regular ion channels, hence higher room temperature conductivities; low-temperature conductivity retention is determined by the bound-water proportion and network elasticity—pine-regenerated cellulose is retained in the pore walls as microfibrils and forms an efficient dynamic hydrogen bond network with PAM, which both binds free water to suppress freezing and maintains channel integrity under ice-crystal stress, so its −40 °C conductivity and retention both rank first and its strain-sensing response is the most robust. Eucalyptus serves as a negative example—the regenerated-cellulose skeleton has poor continuity and thin, brittle walls, and both mechanical performance and room temperature channel connectivity deteriorate, demonstrating that the continuity of the regenerated-cellulose skeleton is a prerequisite for double-network synergistic reinforcement. This rule is consistent with the results of the cellulose/sodium alginate double-network gel electrolytes reported by Zhou et al. [56]: a multi-channel structure with synergistic hydrogen bonding and metal–ion coordination networks can reduce zinc dendrite growth and enhance the ionic conductivity (6.30 mS·cm^−1^) and ion transference number (t^+^ = 0.66). In summary, cotton, bagasse and pine fibers represent three strategies of “room temperature-conductivity priority”, “strength–conductivity balance” and “low-temperature and deformation-adaptation priority”, respectively, and fiber selection should be oriented toward the target application conditions.

## 3. Conclusions

In this study, a series of cellulose/PAM double-network conductive hydrogels were fabricated by in situ polymerization of acrylamide in an AlCl_3_/ZnCl_2_ aqueous system using four bleached pulp fibers (eucalyptus, pine, bagasse, and cotton). Due to differences in DP and dissolution–regeneration states among the four fibers, the resulting regenerated cellulose exhibited distinct pore wall architectures and hydrogen bonding networks, which significantly influenced the antifreezing performance, ionic conductivity, and mechanical properties of the hydrogels. Specifically, the cotton-based gel (highest DP) achieved the best room temperature tensile properties (strength of 105.25 kPa and elongation at break of 1566%) and ionic conductivity (50.53 mS·cm^−1^); the pine-based gel maintained the optimal conductivity at −40 °C (0.83 mS·cm^−1^, 5.1% retention); and the bagasse-based gel showed a 52% increase in tensile strength after freeze–thaw cycles, demonstrating superior freeze–thaw durability. This work establishes material-level structure–property correlations between fiber raw material characteristics and the overall gel performance, providing an experimental basis for fiber selection for antifreezing conductive hydrogels. Device-level validation remains to be completed in future work.

## 4. Materials and Methods

### 4.1. Materials

Bleached kraft eucalyptus pulp, bleached kraft pine pulp, bleached bagasse pulp, and cotton linter pulp boards were commercially obtained from Dalian Yangrun Trading Co., Ltd. (Dalian, Liaoning, China) and used as received without further purification. Anhydrous zinc chloride (ZnCl_2_, ≥98%, Sigma-Aldrich, St. Louis, MO, USA), anhydrous aluminum chloride (AlCl_3_, 99%, Sigma-Aldrich, St. Louis, MO, USA), acrylamide (AM, ≥99.0%, Chengdu Kelong Chemical Co., Ltd., Chengdu, China), N,N′-methylenebisacrylamide (MBAA, 98%, Hangzhou Lanbo Industrial Co., Ltd., Hangzhou, China), and ammonium persulfate (APS, analytical grade, Sinopharm Chemical Reagent Co., Ltd., Shanghai, China) were used as received without further purification. Cupriethylenediamine (CED) solution (analytical grade) was purchased from Shanghai Taixuan Industrial Co., Ltd. (Shanghai, China). Deionized water was used throughout all experiments.

### 4.2. Preparation of the Conductive Hydrogels

A series of polyacrylamide double-network conductive hydrogels modified with different plant fibers were constructed by in situ polymerization via thermally initiated free-radical polymerization (Figure 7). The procedure was as follows: 27.24 g of ZnCl_2_ and 2.50 g of anhydrous AlCl_3_ were weighed and dissolved in 15 mL of deionized water under magnetic stirring until the salts were completely dissolved. Then 0.40 g of bleached pulp board (torn into pieces of ca. 5 mm) was added, and the mixture was heated in a 65 °C water bath to evaporate excess water until the water content decreased to 8.5 mL (ca. 16 h), during which the cellulose gradually dissolved and the system became transparent and homogeneous. Subsequently, 25 g of acrylamide (AM) monomer and 40 mg of N,N′-methylenebisacrylamide (MBAA) crosslinker were added and stirred until completely dissolved, followed by the addition of 20 mg of ammonium persulfate (APS) as the initiator; deionized water was added to bring the total mass to 100 g. The solution was poured into a polytetrafluoroethylene mold and polymerized at 60 °C for 4 h, yielding four fiber-modified zinc-ion-conductive polyacrylamide hydrogels, designated as Zn-PAM/E (eucalyptus), Zn-PAM/P (pine), Zn-PAM/S (bagasse) and Zn-PAM/C (cotton), respectively. Two control samples were prepared: (1) pure PAM hydrogel—no pulp board or metal salts, with all other steps identical; (2) Zn-PAM hydrogel—with ZnCl_2_ and AlCl_3_ but without pulp board, with all other steps identical. All samples were stored in sealed containers at room temperature before use. It should be noted that “Zn-PAM” in the sample designations refers to the PAM gel containing the ZnCl_2_/AlCl_3_ dual metal salts.

The ZnCl_2_/AlCl_3_ ratio and total salt loading were fixed at the composition reported to efficiently dissolve cellulose in this dual-salt system [23], so as to isolate the fiber-source variable in this comparative study; the interactions among formulation parameters (salt ratio, cellulose loading, monomer content) and their systematic optimization will be addressed in future work.

### 4.3. Characterization Methods

#### 4.3.1. Characterization of Raw Materials

The surface morphology of the four pulp fibers was observed using a scanning electron microscope (SEM, S-3400N, Hitachi, Tokyo, Japan) after gold sputtering. FT-IR spectra of the pulp boards were collected at room temperature on a Fourier-transform infrared spectrometer (Bruker Vertex 80v, Bruker, Ettlingen Germany) over the range of 4000–400 cm^−1^ in attenuated total reflection (ATR) mode at a resolution of 4 cm^−1^ with 32 accumulated scans; the lateral order index was calculated as the absorbance ratio of the crystalline band at 1428 cm^−1^ to the amorphous band at 896 cm^−1^, LOI = A_1428_/A_896_ [33]. The crystalline structure of the pulp boards was analyzed using an X-ray diffractometer (XRD-6100, Shimadzu, Kyoto, Japan; Cu Kα radiation, λ = 0.15406 nm, operated at 40 kV and 40 mA) over 2θ = 5–60° with a step size of 0.02° at a scanning rate of 5°/min; CrI was calculated according to the Segal empirical formula [36]:

(1) $$CrI=\frac{I_{002}-I_{am}}{I_{002}}\times 100$$

where I_002_ is the diffraction intensity of the (002) crystal plane at 2θ ≈ 22.5°, and I_am_ is the valley intensity of the amorphous region between 2θ = 18° and 19°.

The thermal stability of the pulp boards was evaluated using a simultaneous thermal analyzer (STA 409 PC, NETZSCH, Selb, Germany) under a nitrogen atmosphere at a heating rate of 10 °C/min over a temperature range from room temperature to 600 °C. DP was determined according to GB/T 1548—2016[57]: the intrinsic viscosity was measured with an Ubbelohde viscometer at (25 ± 0.1) °C using a cupriethylenediamine (CED) solution as the solvent and converted to DP according to the standard formula (DP = 1.65[η], where [η] is the intrinsic viscosity in mL/g).

#### 4.3.2. Characterization of the Hydrogels

The hydrogel samples were freeze-dried and gold-sputtered, and their cross-sectional morphology was observed using a scanning electron microscope (S-3400N, Hitachi, Tokyo, Japan). FT-IR spectra of the freeze-dried gels were collected on a Fourier-transform infrared spectrometer (Bruker Vertex 80v, Bruker, Ettlingen Germany) over the range of 4000–400 cm^−1^. The condensed-state structure of the freeze-dried gels was analyzed using an X-ray diffractometer (XRD-6100, Shimadzu, Kyoto, Japan; Cu Kα radiation) over 2θ = 5–90° at a scanning rate of 5°/min; to compare the scattering differences among the gels, all patterns were normalized in the 2θ = 45–55° region and subtracted using the blank Zn-PAM pattern as the background.

### 4.4. Mechanical Tests

Mechanical tests were performed using a texture analyser (TA-XT plus, Stable Micro Systems, Godalming, UK). Tensile tests: rectangular specimens (40 mm long × 5 mm wide × 2 mm thick) with a gauge length of 10 mm were stretched at 2 mm/s; the elastic modulus was taken as the slope of the linear fit over the 5–25% strain interval, the tensile strength and elongation at break were taken as the peak stress and its corresponding strain, and the toughness was taken as the integrated area under the stress–strain curve up to fracture [58]. Compression tests: cylindrical specimens (15 mm in diameter × 10 mm in height) were compressed at 2 mm/s to 70% strain; the compressive modulus was taken as the slope of the linear fit over the 5–20% strain interval, and the compressive stress at 70% strain (σ_70_) was recorded. Cyclic compression tests: 20 consecutive cycles at 70% strain, with the stress retention calculated by normalizing to the σ_70_ of the first cycle as 100%. Cyclic tensile tests: 20 consecutive cycles at 200% strain, with the relative resistance change recorded synchronously. Low-temperature compression tests: samples were frozen to −30 °C in a condensation device using −40 °C ethanol as the cooling medium, held for 2 h, and then removed and immediately subjected to compression tests with the parameters described above. Freeze–thaw cycling: sealed samples were placed in the cold air of the above condensation device at −40 °C, with each cycle consisting of freezing for 8 h and thawing at room temperature for 2 h, for a total of five cycles, followed by tensile tests as described above. For each group, at least three independently prepared specimens (n ≥ 3) were tested, and representative curves and values are reported.

### 4.5. Electrochemical Tests

EIS was performed using an electrochemical workstation (CHI660, Shanghai Chenhua Instrument Co., Ltd., China): a gel sheet (20 mm × 20 mm × 2 mm) was sandwiched between two stainless-steel blocking electrodes, and the impedance was recorded over a frequency range of 0.1–100,000 Hz with a perturbation amplitude of 5 mV and a quiet time of 2 s; measurements were performed at 20, 0, −20, and −40 °C, respectively. The bulk resistance R_b_ was obtained by extrapolating the high-frequency linear segment of the Nyquist plot to the intercept on the real axis, and the ionic conductivity was calculated as:

(2) $$\sigma =\frac{L}{R_{b}\cdot S}$$

where L is the sample thickness (0.2 cm) and S is the electrode contact area (4 cm^2^). For each group, at least three independently prepared specimens (n ≥ 3) were tested, and representative values are reported.

Strain-sensing tests: both ends of the gel were connected to electrodes and fixed on the texture analyser; the gel was stretched over a strain range of 0–150% while the relative resistance change ΔR/R_0_ was recorded synchronously, and the relative resistance value was calculated by the formula:

(3) $$\frac{∆R}{R_{0}}=\frac{R-R_{0}}{R_{0}}$$

where R_0_ is the initial resistance at zero strain, and R is the resistance under the applied strain. The gauge factor was calculated as:

(4) $$GF=d(∆R/R_{0})/d_{\varepsilon }$$

where ε is the applied strain. GF was taken as the slope of the piecewise-linear fit of the ΔR/R_0_–ε curve.

The apparent activation energy (Ea) for ion transport was obtained by fitting the temperature-dependent ionic conductivities (20, 0, −20, and −40 °C) to the Arrhenius equation:

(5) $$\ln\sigma =\ln\sigma_{0}-\frac{E_{a}}{R_{T}}$$

where σ is the ionic conductivity at the absolute temperature T (K), σ_0_ is the pre-exponential factor, and R is the gas constant (8.314 J·mol^−1^·K^−1^). Ea was calculated as Ea = −k·R × 1000, where k is the slope of the linear fit of ln σ versus 1000/T (Figure S2), and the fitted values are listed in Table S2.

### 4.6. Statistical Analysis

All measurements were performed on at least three independently prepared specimens (n ≥ 3). Given the limited number of replicates, no statistical significance testing was performed; representative data were reported, and differences between groups were discussed as observed trends. Data processing, curve fitting, and plotting were performed using OriginPro 2024 (OriginLab Corporation, Northampton, MA, USA).

## Figures and Tables

**Figure 2:**
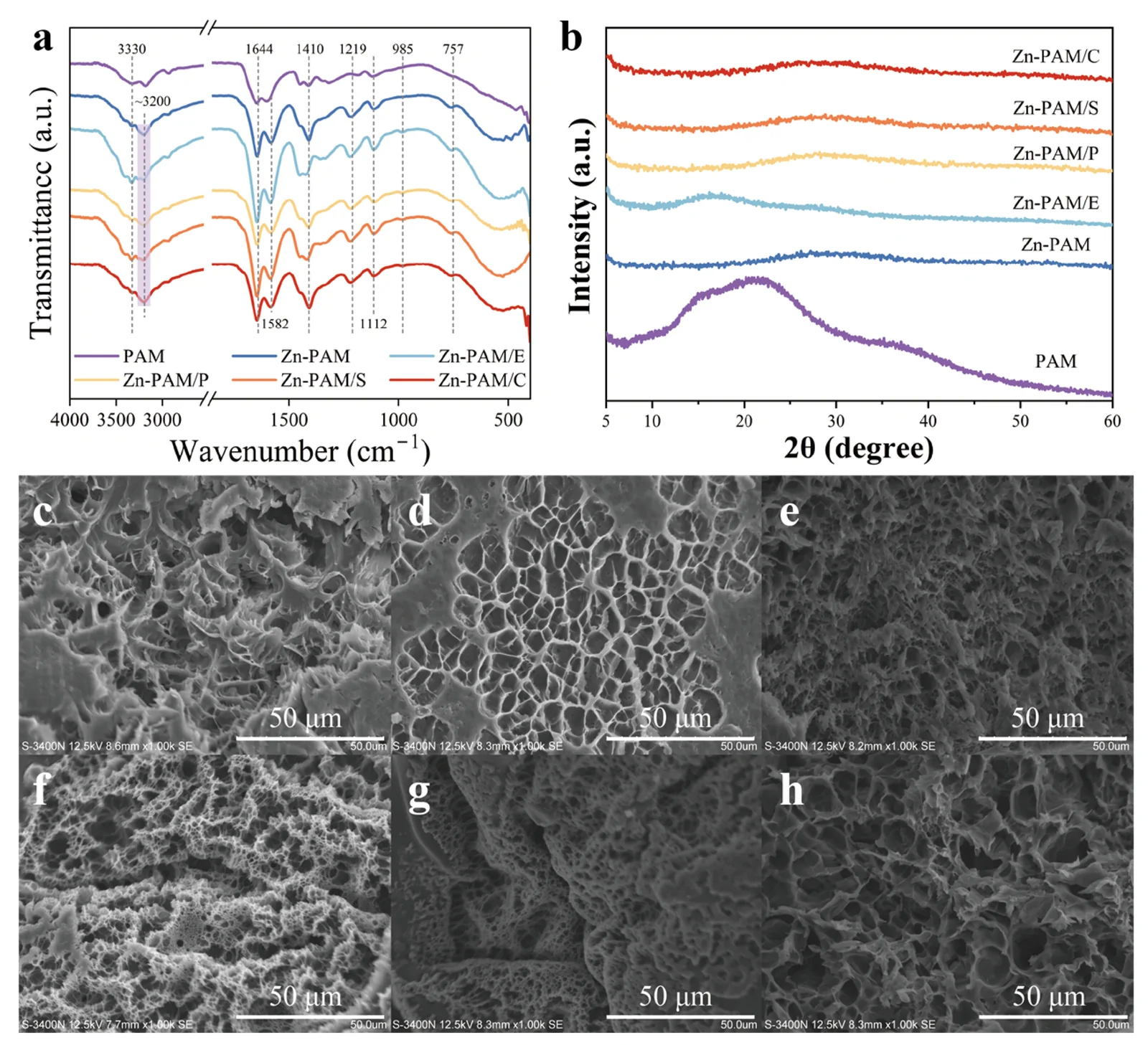
Chemical structure, crystalline characteristics, and cross-sectional morphology of PAM-based hydrogels. (**a**) FT-IR spectra. (**b**) XRD patterns of PAM, Zn-PAM and plant fiber-modified hydrogels. (**c**–**h**) Cross-sectional SEM images of freeze-dried PAM, Zn-PAM, Zn-PAM/E, Zn-PAM/P, Zn-PAM/S and Zn-PAM/C hydrogels, respectively (Scale bar: 50 μm).

**Figure 3:**
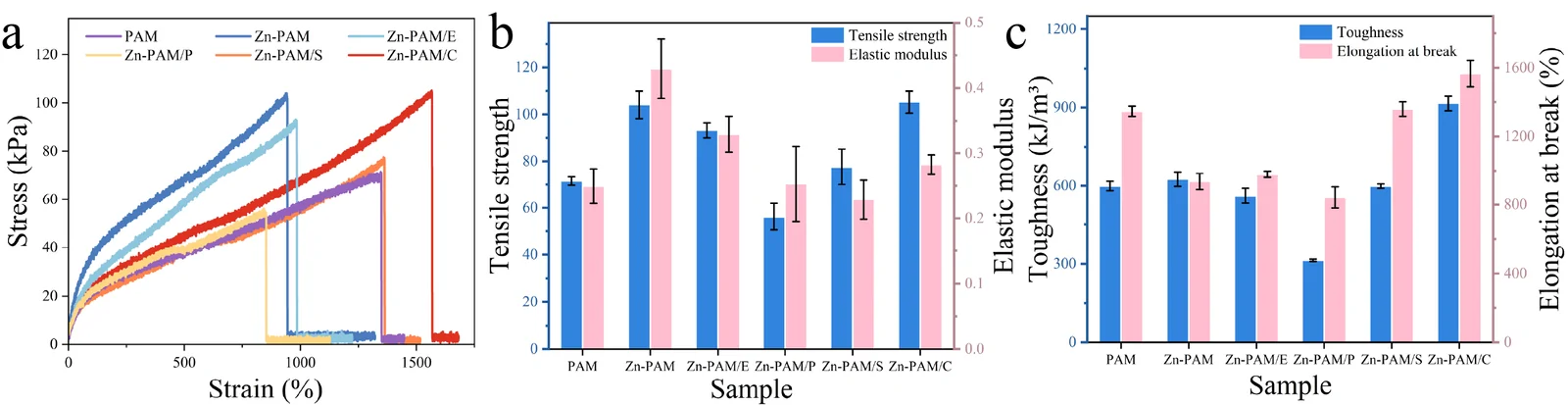
Tensile properties of PAM, Zn-PAM, and plant-fiber-reinforced Zn-PAM/X (X = E, P, S, C) hydrogels at room temperature: (**a**) representative tensile stress–strain curves; (**b**) tensile strength and elastic modulus; (**c**) toughness and elongation at break.

**Figure 4:**
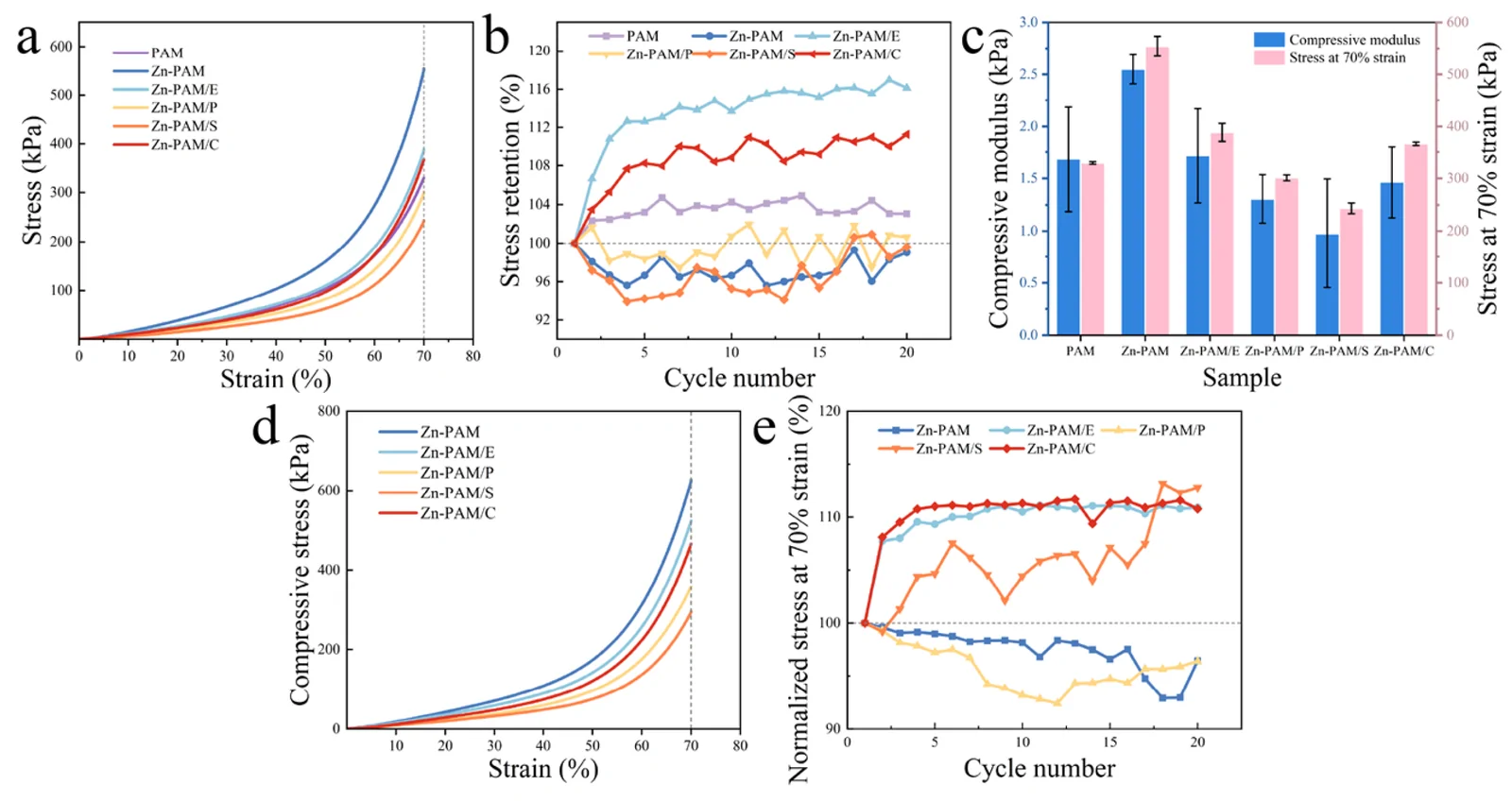
Compressive properties of the hydrogels: (**a**) compressive stress–strain curves at room temperature (70% strain); (**b**) normalized stress retention over 20 cyclic compressions at 70% strain (room temperature); (**c**) compressive modulus and stress at 70% strain; (**d**) compressive stress–strain curves at −30 °C (pure PAM is not included because the stress exceeded the load cell limit); (**e**) normalized stress retention over 20 cyclic compressions at −30 °C (70% strain).

**Figure 5:**
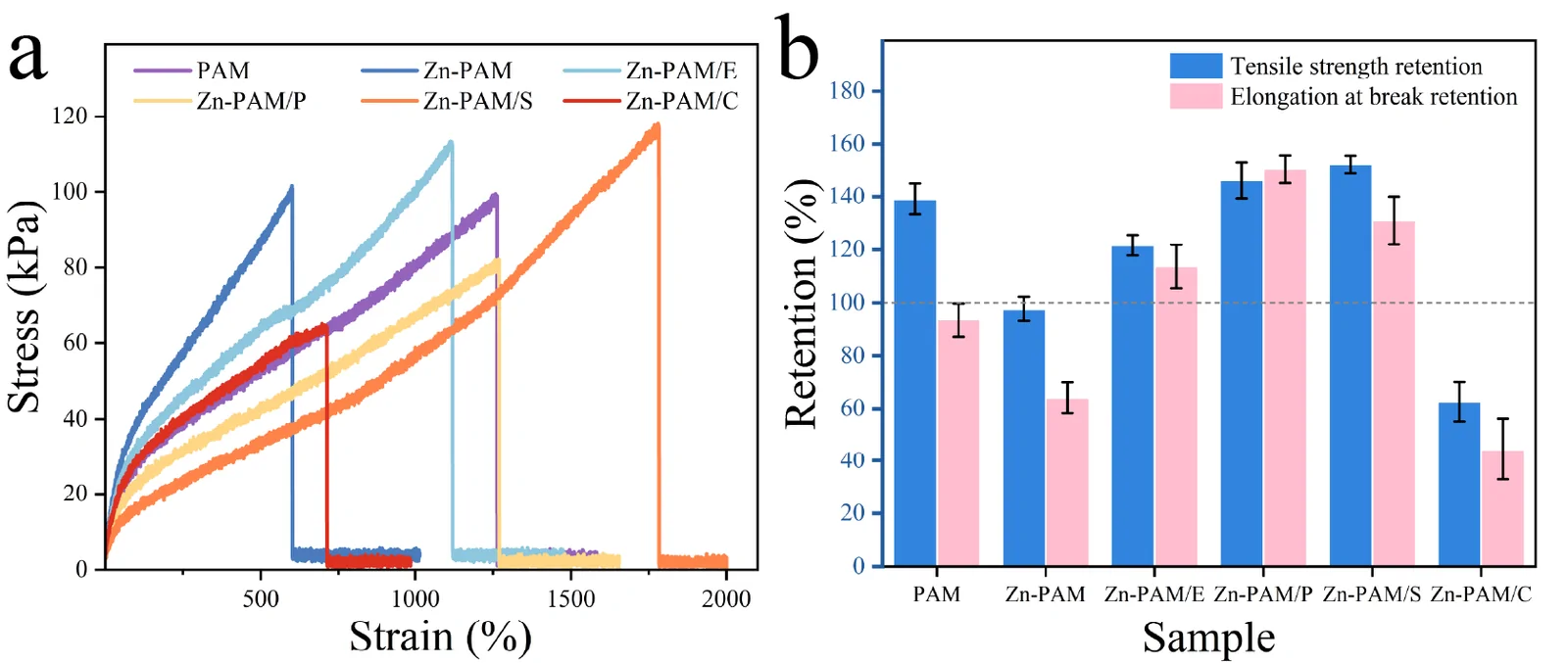
Tensile performance of the hydrogels after freeze–thaw treatment and recovery to room temperature: (**a**) representative tensile stress–strain curves after five freeze–thaw cycles at −40 °C; (**b**) retention of tensile strength and elongation at break relative to the pristine hydrogels.

**Figure 6:**
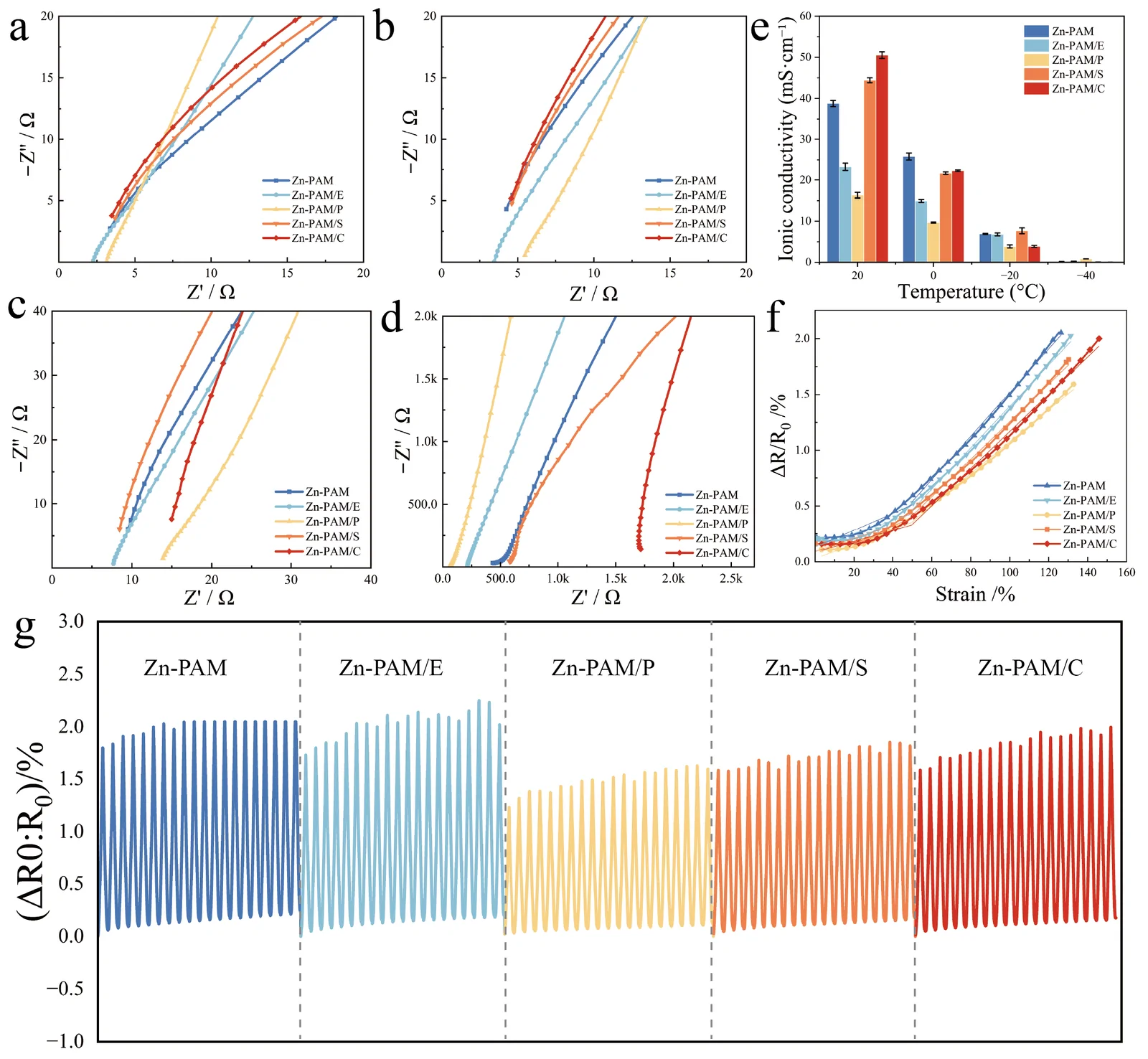
Visual conductivity demonstration, low-temperature ionic conduction characteristics, and strain sensing properties of PAM-based hydrogels. (**a**–**d**) AC impedance spectra of hydrogels at different temperatures. (**e**) Ionic conductivity of hydrogels at various temperatures. (**f**) Relative resistance variation in hydrogels as a function of tensile strain. (**g**) Resistance stability of hydrogels under cyclic tensile loading.

**Figure 7:**
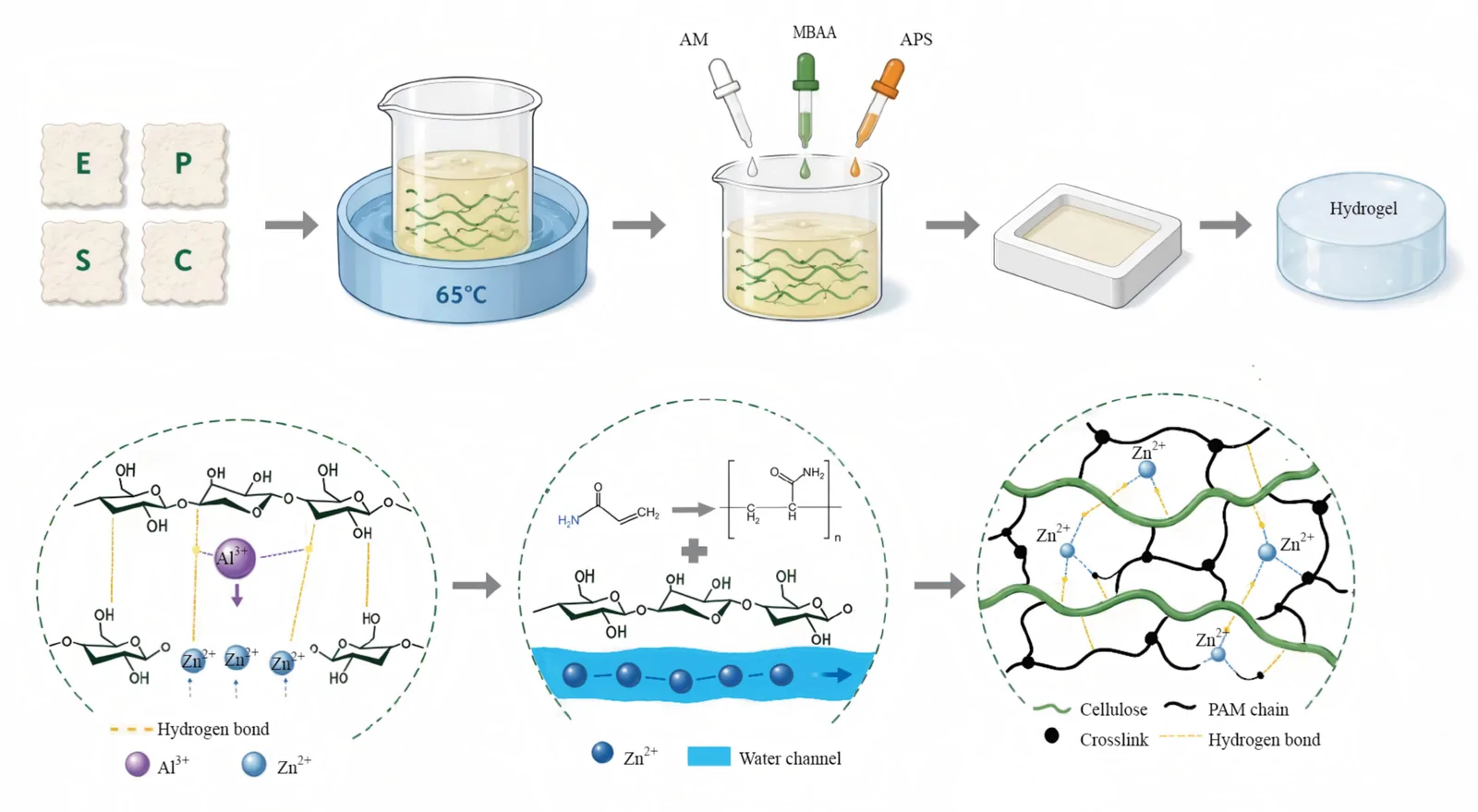
Schematic illustration of the preparation and synergistic ZnCl_2_/cellulose reinforcement mechanism of the antifreezing conductive PAM-based hydrogels.

**Table 1:** Basic properties of the four plant fiber raw materials.

Sample	DP	CrI (%)
Eucalyptus pulp	1082	79.1
Pine pulp	1356	78.6
Bagasse pulp	914	75.2
Cotton pulp	2327	88.2

**Table 3:** Two-segment linear-fitting results of relative resistance change (ΔR/R_0_) versus tensile strain for the hydrogels (20th cycle).

Sample	Strain Regime	Gauge Factor (GF) *	R^2^
Zn-PAM	0–50%	0.759	0.8857
	>50%	1.956	0.9969
Zn-PAM/E	0–50%	0.595	0.8254
	>50%	1.857	0.9966
Zn-PAM/P	0–50%	0.655	0.9198
	>50%	1.436	0.9965
Zn-PAM/S	0–50%	0.586	0.8443
	>50%	1.686	0.9966
Zn-PAM/C	0–50%	0.492	0.8217
	>50%	1.667	0.9957

* GF was determined as the slope of the linear fit of ΔR/R_0_ versus strain in each regime, where ΔR/R_0_ = (R − R_0_)/R_0_ and R_0_ is the initial resistance at zero strain. R^2^ is the coefficient of determination of the linear fit.

## Data Availability

Data will be made available upon request.

## Supplementary Materials

The following supporting information can be downloaded at https://www.mdpi.com/article/10.3390/gels12100865/s1: Figure S1: XRD difference patterns of the four fiber-modified hydrogels (ΔI = I_sample − I_Zn-PAM). All patterns were normalized in the 2θ = 45°−55° region before subtracting the blank Zn-PAM pattern as the background. The weak excess scattering in the 2θ = 19°–22° region (intensity order: Zn-PAM/C > Zn-PAM/S > Zn-PAM/P) is assigned to a small amount of cellulose-II short-range order in the regenerated cellulose; Figure S2: Arrhenius plots (ln σ versus 1000/T) of the ionic conductivities of Zn-PAM and the four fiber-modified hydrogels over the temperature range of 20 to −40 °C. Solid lines are linear fits to all four data points; the downward deviation at −40 °C arises from the onset of partial freezing. The fitted apparent activation energies and correlation coefficients are listed in Table S2; Table S1: Formulation matrix of all hydrogel samples (total mass of each formulation: 100 g; polymerization at 60 °C for 4 h); Table S2: Apparent activation energies (Ea) for ion transport obtained by fitting the temperature-dependent ionic conductivities (20, 0, −20 and −40 °C) to the Arrhenius equation σ = σ_0_·exp(−Ea/RT); Figure S3: Linear sweep voltammetry (LSV) curves of the five salt-containing hydrogels measured in a two-electrode stainless-steel cell (0 → +2.5 V, scan rate 1 mV s^−1^). The dashed line marks the threshold current density (0.1 mA cm^−2^) used to define the anodic stability limit. The current plateau above ca. 1.6 V corresponds to the instrument range limit (10 mA), not to a sample property; Table S3: Anodic electrochemical stability limits of the five salt-containing hydrogels obtained from the LSV curves in Figure S3 (defined as the voltage at which the current density reached 0.1 mA cm^−2^).

## Abbreviations

The following abbreviations are used in this manuscript:

AM	Acrylamide
APS	Ammonium Persulfate
CED	Cupriethylenediamine
CrI	Crystallinity Index
DP	Degree of Polymerization
DTG	Derivative Thermogravimetric
EIS	Electrochemical Impedance Spectroscopy
FT-IR	Fourier-Transform Infrared
GF	Gauge Factor
LOI	Lateral Order Index
MBAA	N,N′-Methylenebisacrylamide
PAM	Polyacrylamide
SEM	Scanning Electron Microscopy
TG	Thermogravimetric
XRD	X-ray Diffraction
