# Supplementary material for: Effect of a Fiber Raw Material Structure on the Properties of Cellulose/Polyacrylamide Double-Network Antifreezing Conductive Hydrogels Prepared in an AlCl3/ZnCl2 Aqueous System
Source: Gels. 2026 Sep 24;12(10):865. doi: 10.3390/gels12100865 (PMC13704065; doi:10.3390/gels12100865)
Supplement: Supplementary file 1 [file gels-12-00865-s001.zip › gels-4533418-supplementary.pdf]

Supplementary Materials

Effect of Fiber Raw-Material Structure on the Properties of Cellulose/Polyacrylamide Double-Network Antifreezing Conductive Hydrogels Prepared in an $AlCl_3/ZnCl_2$ Aqueous System

Xiaowei Zhuang, Shuqi Guo, Yue Liu, Zhijun Wu, Yongshun Feng, Hui Qiao, Guoqiang Zhu and Xin Pan

Figure S1

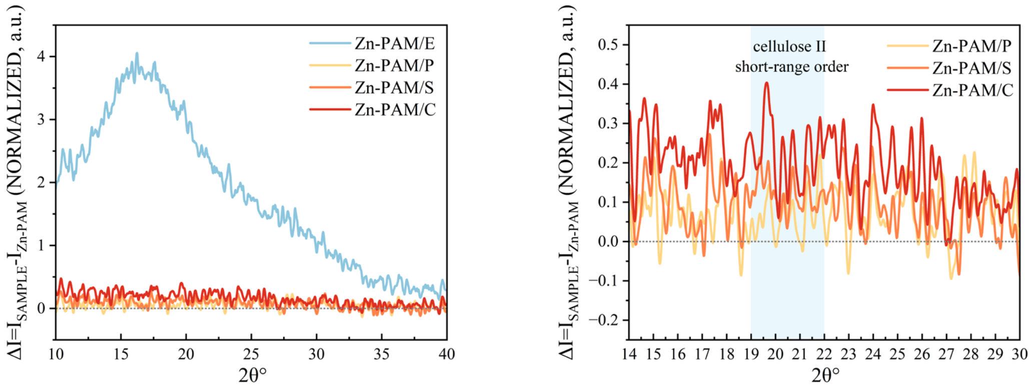

(a) **(b)**

Figure S1. XRD difference patterns of the four fiber-modified hydrogels (ΔI = I_sample − I_Zn-PAM). All patterns were normalized in the 2θ = 45°–55° region before subtracting the blank Zn-PAM pattern as the background. The weak excess scattering in the 2θ = 19°–22° region (intensity order: Zn-PAM/C > Zn-PAM/S > Zn-PAM/P) is assigned to a small amount of cellulose-II short-range order in the regenerated cellulose.

Figure S2

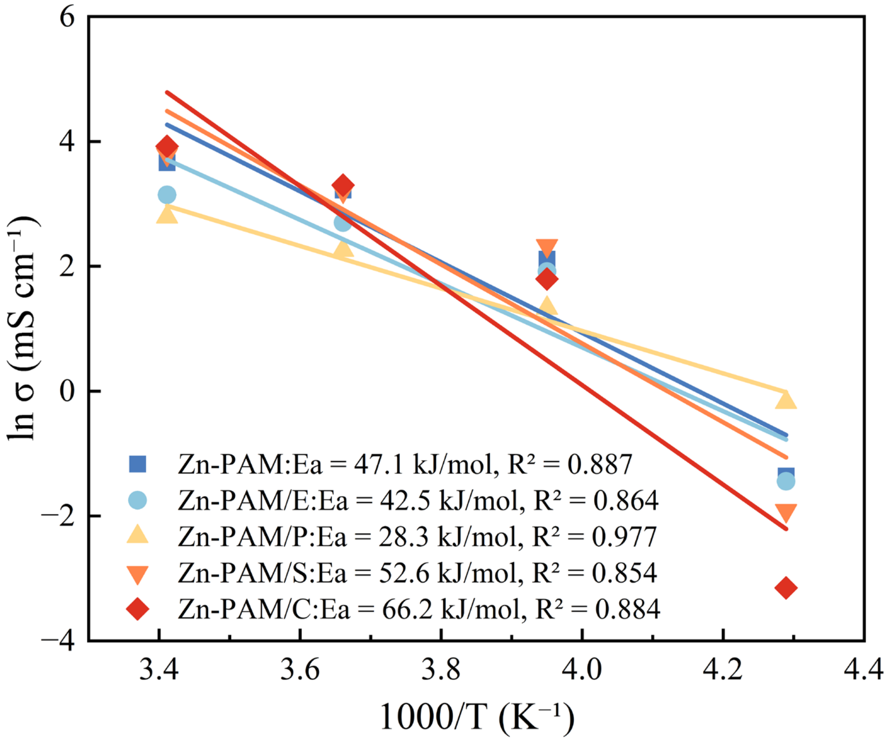

Figure S2. Arrhenius plots (ln σ versus 1000/T) of the ionic conductivities of Zn-PAM and the four fiber-modified hydrogels over the temperature range of 20 to −40 °C. Solid lines are linear fits to all four data points; the downward deviation at −40 °C arises from the onset of partial freezing. The fitted apparent activation energies and correlation coefficients are listed in Table S2.

Table S1

Table S1. Formulation matrix of all hydrogel samples (total mass of each formulation: 100 g; polymerization at 60 °C for 4 h).

Sample	Pulp source	Pulp (g)	$ZnCl_2$ (g)	$AlCl_3$ (g)	AM (g)	MBAA (mg)	APS (mg)	Deionized water (g)
PAM	—	0	0	0	25	40	20	to 100
Zn-PAM	—	0	27.24	2.50	25	40	20	to 100
Zn-PAM/E	Bleached eucalyptus pulp	0.40	27.24	2.50	25	40	20	to 100
Zn-PAM/P	Bleached	0.40	27.24	2.50	25	40	20	to 100

	pine pulp							
Zn-PAM/S	Bleached bagasse pulp	0.40	27.24	2.50	25	40	20	to 100
Zn-PAM/C	Cotton linter pulp	0.40	27.24	2.50	25	40	20	to 100

Table S2

Table S2. Apparent activation energies (Ea) for ion transport obtained by fitting the temperature-dependent ionic conductivities (20, 0, −20 and −40 °C) to the Arrhenius equation $\sigma = \sigma_0 \cdot \exp(-Ea/RT)$.

Sample	Ea (full range, 20 → −40 °C) ($kJ \cdot mol^{-1}$)	R^2	Ea (non-frozen range, 20 → −20 °C) ($kJ \cdot mol^{-1}$)	R^2
Zn-PAM	47.1	0.887	24.0	0.959
Zn-PAM/E	42.5	0.864	19.0	0.987
Zn-PAM/P	28.3	0.977	22.6	0.987
Zn-PAM/S	52.6	0.858	22.7	0.995
Zn-PAM/C	66.2	0.884	33.1	0.964

Note: the ln σ–1000/T curves deviate downward at −40 °C owing to the onset of partial freezing; the full-range values are therefore apparent parameters. The non-frozen-range fits (20 → −20 °C) exclude the −40 °C point.

Electrochemical stability (LSV): The electrochemical stability of the five salt-containing hydrogels was evaluated by linear sweep voltammetry (LSV) on a CHI660E electrochemical workstation (Shanghai Chenhua Instrument Co., Ltd., China) in a two-electrode configuration, in which a hydrogel sheet (20 × 20 × 2 mm) was sandwiched between two stainless-steel plates. The potential was scanned from 0 to +2.5 V at 1 mV s^{-1}, and the anodic stability limit was defined as the voltage at which the current density (normalized to the 4 cm^2 electrode contact area) reached 0.1 mA cm^{-2}. At least three specimens ($n \geq 3$) were measured for each group and representative curves are shown. As shown in Figure S3 and Table S3, the five gels exhibit anodic stability limits of 1.23–1.34 V, with the blank Zn-PAM gel showing the highest value (1.34 V) and the four fiber-modified gels ranging from 1.23 to 1.33 V, indicating that the high-concentration $ZnCl_2/AlCl_3$ salt environment endows the gels with stable electrochemical windows suitable for low-voltage flexible electronics.

Figure S3

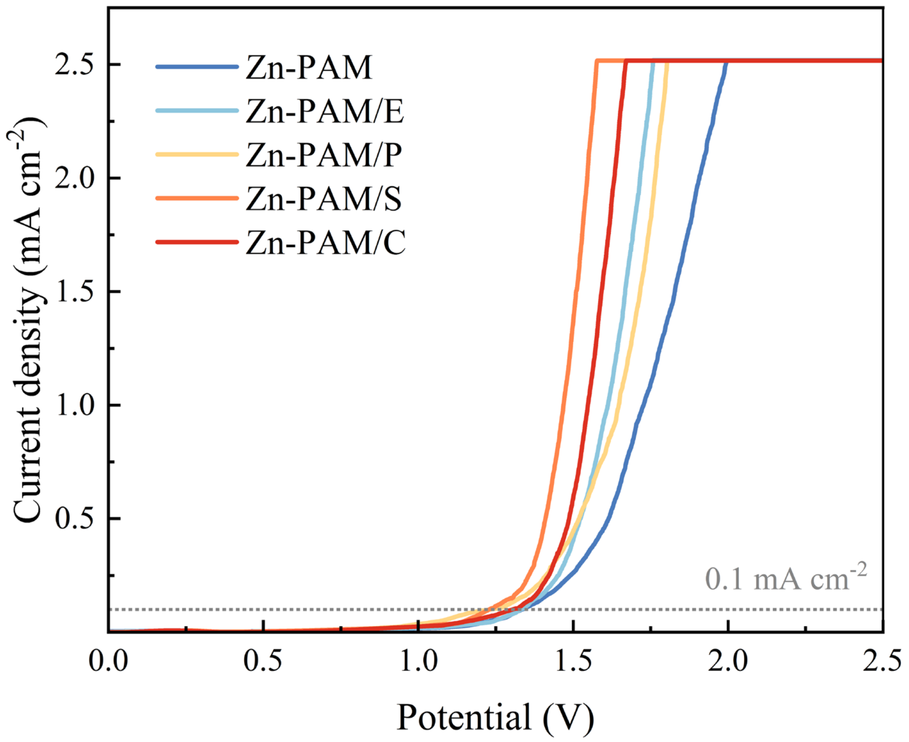

Figure S3. Linear sweep voltammetry (LSV) curves of the five salt-containing hydrogels measured in a two-electrode stainless-steel cell (0 → +2.5 V, scan rate 1 mV s^{-1}). The dashed line marks the threshold current density (0.1 mA cm^{-2}) used to define the anodic stability limit. The current plateau above ca. 1.6 V corresponds to the instrument range limit (10 mA), not to a sample property.

Table S3

Table S3. Anodic electrochemical stability limits of the five salt-containing hydrogels obtained from the LSV curves in Figure S3 (defined as the voltage at which the current density reached 0.1 mA cm^{-2}).

Sample	Anodic stability limit (V)
Zn-PAM	1.34
Zn-PAM/E	1.33
Zn-PAM/P	1.23
Zn-PAM/S	1.23
Zn-PAM/C	1.30
